# The Role of Ambiguity Tolerance and Enthusiasm on Chinese University Teachers’ Burnout

**DOI:** 10.3389/fpsyg.2022.910598

**Published:** 2022-06-23

**Authors:** Yan Yang, Juan Xie

**Affiliations:** ^1^School of Innovation and Entrepreneurship, Hunan Institute of Technology, Hengyang, China; ^2^School of Computer Science and Engineering, Hunan Institute of Technology, Hengyang, China

**Keywords:** ambiguity tolerance, enthusiasm, teachers’ burnout, Chinese university teachers, academic

## Abstract

Educators face numerous vague conditions in their daily practice and they must manage students with diverse characters that lead to burnout. In addition, tolerance of ambiguity is a term used by educators as the capability to control new, complicated or insoluble situations. Moreover, educator enthusiasm and its effect on instruction was neglected. And due to the essential function that teachers’ burnout plays in the efficiency of their achievement in the scholastic cycle, the present study inspected whether the above-mentioned constructs such as teachers’ ambiguity tolerance and enthusiasm can influence their burnout on one hand and on the other hand if these constructs can predict the teachers’ burnout or not. For the objective of the study, a group of 495 female and male Chinese university teachers in 18 provinces of China were asked to fill out the three scales, namely, teacher enthusiasm, ambiguity tolerance, and burnout. The primary results of the research, achieved through running Spearman Rho, specify that there are constructive relationships between ambiguity tolerance, enthusiasm and by employing multiple regression analysis; it is revealed that both variables, namely ambiguity tolerance, enthusiasm were the predictors of teachers’ burnout, while ambiguity tolerance was a better predictor. Accordingly, based on these findings, it can be concluded that both of these factors affect teachers’ burnout in the teaching process. In addition, this study can provide additional implications for academic scholars and experts in academic settings.

## Introduction

Among intense loads of work, country obligations, and difficulty in satisfying students’ requirements, a majority of educators’ reports in the primary and secondary schools indicated great degrees of stress (Herman et al., 2020; Wang, 2021). Indeed, tension and stress influence the bodily well-being and mental status of thought, consisting of the experience of low self-respect, dejection, irascibility, hopelessness, apprehension, bitterness, lack of expert incentive, sense of career insecurity, and lack of efficiency in the overall education duties (Aybas et al., 2015; Sneha and Maheswari, 2020). The primary stressing factors mentioned within the literature include the learners’ lack of passion and lack of order; lack of conversation, lack of structure, and authoritarianism exerted by each the coordination and the school route, policies and methods that lead to disempowerment, colleagues’ individualism and competition, and not enough salaries (Skaalvik and Skaalvik, 2017) and usually, the ongoing school fact obliges educators to deliver lessons in poor conditions, a social help that exceeds those concerning teaching (Prilleltensky et al., 2016). As a notion associated with educators’ feelings, anxiety has been demonstrated as a widespread issue in various academic environments that, when felt ceaseless, results in burnout (Fathi et al., 2021). Because of the stressful working conditions, educators normally encounter “burnout” as survey information indicates, education is regarded as a career involving “intense tension,” and almost 25% of school educators consider education as extraordinarily stressful (Pishghadam et al., 2019; Wang et al., 2022). Indeed, recently, attention has been paid to the burnout phenomenon, especially in careers associated with human services. Being exhausted from hard work and putting high energies from themselves, people encounter burnout syndrome (Küçükoğlu, 2014) which features affective fatigue, or a lack of enthusiasm for instructing; losing personality, or detachment from learners and work; and a loss of individual success (Schaufeli et al., 2009).

Researchers and scholars are paying growing attention to burnout in educators because its intensity among academic experts made instruction a career involving high risk regarding the growth of this syndrome (Guidetti et al., 2017). Its incidence influences the academic and the social settings, intervention of the success of pedagogical outcomes. Besides, the education quality is related to decreased satisfaction with the job, decreased feeling of educational effectiveness, the purpose to give up education, absence, and low-quality individual life (Rabasa et al., 2016). Burnout is characterized as a mounting response to long-term work-related inconveniences, described by indications of emotional exhaustion, depersonalization, and decreased individual success (Maslach and Leiter, 2016). Ever since its appearance, burnout has been recognized as a career-relevant risk to individual-directed professions that require a high degree of individual proximity (Leiter and Maslach, 2000). Educators’ burnout is significant for studies because it affects the supply of successful teaching. Therefore, burnout can have a widespread impact on the well-being of school educators and learners. In the present environment of high-stakes tests and elevated educator responsibility and examination, teachers might encounter anxiety as a part of their work. Although burnout and anxiety are regularly utilized alternately at times, anxiety could ultimately result in career burnout (Skaalvik and Skaalvik, 2017). Another mentioned symptom of burnout is the negative viewpoint, namely, restlessness, less toleration, and a feeling of frustration in the workplace or life (Sneha and Maheswari, 2020).

Efficient educator traits, namely, incentive, close relationship, or enthusiasm are strongly relevant to a large number of variables like success, incentive, and interest. Indeed, studies have confirmed that the manner learners understand their educators’ behavior affects their educational success, feelings, viewpoint, and incentive (Becker et al., 2014; Misbah et al., 2015). Educator enthusiasm is among the affective elements that elevate the efficacy of instruction and education (Cui et al., 2017; Hooda and Annu, 2018). Furthermore, enthusiastic teachers seem to be more contented and healthier and are more influential in education (Lazarides et al., 2019). Educators who experience burnout possibly do not engage in the class or cannot motivate the students, they teach with lower enthusiasm and creativity, and they have a lower education commitment which directly affects students’ learning. This is because it is the educators’ role to aid students to find methods of improving their next degree of understanding (Williams and Burden, 2000).

In a conceptual sense, educator enthusiasm, through which educators transmit their great level of energy and thrill to the learners, and which is demonstrated by nonverbal expressions, is regarded as one of the primary attributes of a successful educator (Baloch and Akram, 2018). One could say that the elements that emphasize transmission and association between the educator and learner, and which demonstrate the standard of instructing service are among the predictors of educator enthusiasm in the cycle of instructing and education (Hotaman and Sahin, 2010). These elements include offering learners feedback, fixing their potential errors, offering adequate reinforcement at the right time, and arranging the instructing and educating positions in a way that will help the involvement of learners. The educator’s capability of controlling the tone, velocity, and volume of his or her voice in class, offering fluent and unforgettable oral articulations by choosing the right words, possessing a constructive and clear facial expression, to have eye contact with the learner, to utilize movements, mimics, and body language in the right way, and to possess energetic and lively manners are among the predictors of educator enthusiasm (Kasalak and Dagyar, 2020).

In learning, the ability to control novel unknown situations without becoming frustrated is called ambiguity tolerance (Chu et al., 2015). In such situations, the individual’s character determines the extent to which this uncertain situation can be successfully tended to (Hancock and Mattick, 2020). The concept of tolerance of ambiguity stems from general psychology and is a personal contrast factor. Throughout the years, the notion has attained substantial interest in the domains of general psychology, clinical psychology, institutional psychology as well as social psychology, and second language studies. Ambiguity tolerance refers to “uncertain situations with ambiguous, imperfect, separated, several, possible, inconsistent, unsure, unstructured, opposite, conflicting, or obscure signs (Chang, 2020). Tolerance of ambiguity is commonly considered as the skill of controlling circumstances that are novel, intricate, and hold issues without a direct solution and these three attributes could also be ascribed to the action of studying a foreign language since the language is new, intricate, and usually without direct solutions for language issues (Dörnyei, 2005). The function of ambiguity tolerance in language education has been vastly accepted by teachers because language educators consistently encounter different ambiguous triggers involving linguistic and cultural problems (Kamran, 2011). Ambiguous and unsure conditions comprise part of educators’ daily work life. The capability to cope with these conditions influences the effectiveness, decisions, and class management of educators. Educators are conscious of their ambiguity tolerance who perceive its possible effect on their minds and conduct in the class can use such knowledge and grow greater effective tactics in the manner of coping with unforeseen situations inside the class setting (Sokolová and Andreánska, 2019).

Even though ambiguity tolerance is presumed to be an essential segment of the career practice (Hammond et al., 2017), there are not enough studies on the function of ambiguity tolerance in educators’ burnout. For example, in research carried out by Iannello et al. (2017), it was discovered that teachers with a great degree of ambiguity intolerance handled ambiguous circumstances more inflexibly, and so, encountered more career anxiety. Moreover, some research that has explored the impact of enthusiasm on burnout is not related to teachers’ domain. As a result, the present paper intends to emphasize teachers’ burnout and conceptualize the roles of two factors, enthusiasm and ambiguity tolerance among Chinese college teachers, and explore whether they can predict teachers’ burnout to lessen their problems in the procedure of education. As a result, the research questions are formulated as follows:

Q1: Is there a possible relationship between Chinese teachers’ ambiguity tolerance, burnout, and enthusiasm.

Q2: Can Chinese university teachers’ ambiguity tolerance and enthusiasm predict their level of burnout?

## Review of the Related Literature

### Teachers’ Burnout

Even though there are numerous meanings for career burnout, Maslach and Leiter (2016) suggested that burnout includes three concepts: emotional exhaustion, depersonalization, and the absence of individual success. First, emotional exhaustion alludes to the outer display of being exhausted. For instance, when educators are tired, they might document feeling exhausted or not having enough energy (Friedman-Krauss et al., 2014). Emotional exhaustion represents the mental condition of a person who has deconstructive feelings about work, feelings of dissatisfaction, hopelessness, pessimism, and exhaustion because they cannot cope with the anxiety and tension associated with performing their assignments and obligations where they work (Maslach, 2003).

Second, depersonalization is the function of building a skeptical demeanor combined with an emotive or physical separation from the job, potentially causing educators to overall ignore the emotive and educational requirements of learners (Sas et al., 2011). The depersonalization of people leads to them having a deconstructive demeanor towards the ones who make up their career circle. It might be anticipated that people who have a sense of depersonalization portray deconstructive manners like withdrawing, feeling lonely, being heartless, becoming strong, being estranged, and being incapable of showing compassion (Leiter and Maslach, 2016). People who encounter emotional exhaustion and feel depersonalized begin to evaluate themselves in a deconstructive way in the job they carry out. People who are incapable of attaining a result from their endeavors might blame themselves for insufficiency, and so, they might feel as though they have not had any achievements (Arens and Morin, 2016).

Third, educators suffering from burnout might feel an absence of individual success as they cannot see the advantages of their endeavors and usually feel incapable (Maslach et al., 2001). Demerouti et al. (2001) expanded the Maslach hypothesis with the Job Demands-Resources Model (JD-R), which proposes that burnout leads to an overabundance of requirements of the worker, as well as minimal assets offered to satisfy these requirements. Galbán (2018) explained the cycle of the emergence of the burnout condition as starting with a disequilibrium between institutional requirements and individual assets resultant from emotional exhaustion in the employee; consequently, depersonalization or coping is encountered, which results in the employee being disappointed and tired that leads to low individual achievement at the workplace due to ineffectiveness when encountering various career inconveniences.

### Teacher Enthusiasm

Educator enthusiasm is attended to in two aspects; that is, instructing enthusiasm and subject enthusiasm (Kunter et al., 2011) is the thrill the educator portrays when instructing. It is characterized as instructing with a lot of enthusiasm, the educator’s joy of instruction, and interconnecting with the learners and associating with them. Subject enthusiasm is connected to an educator’s interest in his or her domain. It could be characterized as educators’ joy of working in their domain, their thrill of doing studies on their domain, their attempt to associate with the learner, and their constructive perspective on their domain (Kasalak and Dagyar, 2020). Constructive feelings of educators with great levels of instructing and subject enthusiasm while presenting their job might allow them to be estranged from deconstructive feelings about their career (Patrick et al., 2003). Kunter et al. (2008) presume that educator enthusiasm is a character attribute portrayed in specific affectively decided manners like joy, thrill, and satisfaction in presenting class activities, attained from educators’ innate inspiration, and constructive demeanor and interest in the course as well as instructing it. Enthusiasm was diagnosed as a critical characteristic of a successful educator and also a predictor of the learning behavior of learners, affective moods, function, and interest (Goetz et al., 2013; Kim and Schallert, 2014).

Educator enthusiasm has two broad conceptualizations, namely, enthusiasm as educational behavior, and enthusiasm as a character feature. The first perspective interprets educator enthusiasm as the efficient education manner quality that affects learners’ performance through showing high degrees of power and interest within the topic and offering the lessons in an active, encouraging method. It is associated with the educator’s clarity and capacity to convey the significance and inner value of the learning content to learners (Kim and Schallert, 2014). The second considers educator enthusiasm as an individual inclination and emotional element. This is conceptualized as a repetitive affection indicating the level of entertainment, exhilaration, and joy that educators usually experience in their career tasks (Kunter et al., 2011). Educator enthusiasm is studied in two aspects, including education enthusiasm and topic enthusiasm (Kunter et al., 2011). Education enthusiasm is the exhilaration that the educator shows all through education, which is described as instruction with high levels of enthusiasm, the educator’s pleasure of instruction, and speaking with the learners and interacting with them. Topic enthusiasm pertains to the educator’s interest in their area of teaching. This might be described as the educator’s pleasure of activity in their area, their exhilaration for researching in their field, their efforts for interacting with the learners, and their positive attitude concerning their fields (Kasalak and Dagyar, 2020).

### Ambiguity Tolerance

Ambiguity tolerance was at first regarded from a socio-psychological viewpoint and characterized through its connection with concepts of ethnocentrism, assertiveness, and autocracy (McLain et al., 2015). Ambiguity intolerance can be defined as a tendency to discern ambiguous circumstances as origins of risk, and ambiguity tolerance can be defined as a tendency to discern ambiguous circumstances as absorbing. In this meaning, ambiguous circumstance alludes to a circumstance in which the absence of adequate signals obstructs people from completely structuring or classifying the circumstance (McLain, 2009). There are three kinds of such a circumstance denoted by their originality, intricacy, or unsolvability. Original circumstances are circumstances in which all signals are unknown. Intricate circumstances are those in which there are too many signals to be regarded. Insolvable circumstances are those in which various signals mean various formats (McLain, 2009).

Ambiguity tolerance is defined by the way people discern and react to ambiguous, unknown, or erratic circumstances or triggers (Arquero and McLain, 2010). Under such circumstances, ambiguity can restrict decision-making and forecast (McLain, 2009). On the one hand, intolerance of ambiguity is thus a dislike of the absence of knowledge; on the other hand, ambiguity tolerance means embracing or even being attracted to ambiguous circumstances (Arquero et al., 2017). Individuals with low levels of ambiguity tolerance are inclined to encounter worry, anxiety, and pain when coming across ambiguous triggers. However, people with high levels of ambiguity tolerance assess unknown and ambiguous triggers as desired and fascinating (Xu and Tracey, 2014). Ambiguity tolerance, as opposed to intolerance, is regularly characterized as an intellectual technique; thus, ambiguity tolerance is associated with other elements like less demand for structure, greater resilience, risk-taking, and less stress or imagination (McLain, 2009). The degree of ambiguity tolerance impacts a person’s decision-making in a circumstance when knowledge is lacking, unattainable, or ambiguous. When encountering an unknown circumstance, people with lower levels of ambiguity tolerance are inclined to act in a manner that lessens the unknown, namely, classifying, naming, or typecasting (Valutis, 2015). Ambiguity tolerance may additionally affect educators’ attitudes toward creative instructing approaches, their decisions in difficult class conditions (for example class and contradiction management, misconduct interferences, and so forth.), or might also affect their viewpoints towards variation within the class (e.g., behaving younger students with particular teaching needs, admitting children from various socio-cultural environments and so on; Sokolová and Andreánska, 2019).

A large number of researchers have found that tolerance for ambiguity can be taken into account as a basic dimension to describe the character of people (Li and He, 2016). Tolerance for ambiguity refers to the situation in which someone faces complicated new conditions and accepts them with no feelings of frustration. Ambiguous conditions are those for which people have no sufficient data. The ability to understand ambiguity in theory and action is called ambiguity tolerance impartially and openly. People who can tolerate ambiguity can enjoy ingenious ways with no mental or affective effects of changeability or ambiguity (Hadley, 2003).

Additionally, Cooke et al. (2013) planned a questionnaire on burnout, flexibility, and tolerance of ambiguity in Australian overall practice registrars. Their results demonstrated that staying away from unpredictability, unwillingness to disclose unpredictability, and stress was associated with greater degrees of burnout. Flexibility was negatively associated with unpredictability evasion, burnout, and unwillingness to disclose unpredictability. Likewise, Kuhn et al. (2009) believed that intolerance of unpredictability, which was established in a worry for unfortunate results, was greatly connected to emotional exhaustion and was the strongest indicator of burnout. It is mentioned that the notions of career fulfillment and educator inspiration, which demonstrate a constructive connection with educator enthusiasm, are associated with educators’ burnout in a deconstructive manner (Kasalak and Dağyar, 2021). Based on the above-mentioned literature review, the current study intends to focus on teachers’ burnout and hypothesize the functions of two factors, enthusiasm and ambiguity tolerance among Chinese teachers, and examine their predictor roles in teachers’ burnout.

## Materials and Methods

### Participants

The participants were 495 teachers including both genders (male = 249; female = 246) with different age groups, ranging from 18 to 62. More than half of them (288/58.1%) were young teachers whose age group was from 18 to 30 and who had gotten different academic qualifications and years of teaching experience. Based on convenience sampling, they were gleaned from different institutes, colleges, and universities in China with the majority in Jiangsu and Zhejiang Province (295/59.6%), and others in some other 17 provinces (198/40%) and 2 municipalities (Beijing, Shanghai) directly under the Central Government. Consent had been given to all participants before they took part in this study. All responses were based on their own willingness.

### Instruments

Corresponding to the determinations of the study, the following instruments were employed:

#### Teachers’ Enthusiasm Questionnaire

A 10-item questionnaire was developed by Dweik and Awajan (2013) for the participants’ answers to the questionnaire. Teachers were supposed to designate their degree of enthusiasm to motivational bases by employing a five-point Likert. In the current study, the scale’s estimated Cronbach’s alpha reliability coefficient was 0.971.

#### Multiple Stimulus Types Ambiguity Tolerance Scale-II

MSTAT-II is designed and proved by McLain (2009) to estimate people’s overall tolerance/intolerance for ambiguity. The scale includes 13 items scored on a 5-point continuous Likert scale starting from 1 (strongly disagree) to 5 (strongly agree). Nevertheless, for items # 1, 2, 3, 4, 5, 6, 9, 11, and 12, which are adversely articulated, scoring should be reversed. People’s low scores portray their dislike of ambiguity, while their high scores portray their interest in ambiguity. In this research, the reliability calculated through Cronbach’s alpha coefficient was 0.957.

#### Maslach Burnout Inventory

MBI is the most widely utilized tool designed and proved by Maslach and Jackson (1981) to estimate people’s degree of burnout. The survey consists of 22 items estimating three subscales of emotional exhaustion, depersonalization, and lessened individual success. The items are rated on a 7-point frequency scale starting from 0 (never) to 6 (every day). Every subject’s total result could range from 0 to 154. On this scale, people’s greater results portray their greater degree of burnout (Maslach and Jackson, 1981). In this paper, the scale’s estimated Cronbach’s alpha reliability coefficient was equal to 0.955.

### Data Collection Procedures

To generalize the results of the study, by distributing a questionnaire online *via* Wenjuanxing (a program to collect the data), data was smoothly collected in February from 32 institutes, colleges, and universities in 18 provinces of China. Teachers had been informed that they should put themselves in normal time rather than in the COVID-19 pandemic when they fill in the questionnaire, which had been translated into the source language (Chinese) by two experts in translation and had been double examined by the researcher’s colleagues before being sent to participants. The whole process lasts for 23 days, from January 26th to February 17th. All participants were instructed on how to fill in the questionnaire with their own devices and how to deal with any problem that they might be encountered in the study. Since they had made no contact with the researcher, there would be no possibility of interest conflict and human intervention. Later, the data was cleansed and carefully checked by the researcher before it was sent to SPSS for further exploration. In the final step, the processed data was used to probe into research questions.

### Data Analysis

The Spearman Rho test was implemented to examine the probable relationship among the key variables of this research. Furthermore, a linear multiple regression analysis was used to answer the second research question to check the predictor role of ambiguity of tolerance and enthusiasm on their burnout.

## Results

The study aims to scrutinize the role of Chinese university teachers’ ambiguity tolerance, burnout, and enthusiasm. To decide on the data analysis, preliminary measurements should be done. The first step is to measure the reliability of the three questionnaires used in this study (Table 1).

**Table 1:** Reliability of the questionnaires.

Questionnaires	Cronbach’s alpha	No. of items
Ambiguity tolerance	0.955	13
Teacher burnout	0.971	22
Enthusiasm	0.957	10

To measure the reliability indices of all three questionnaires, the process of calculation was repeated three times and the outputs of Cronbach’s alpha revealed that the Ambiguity Tolerance questionnaire (*r* = 0.95), Teachers’ burnout questionnaire (*r* = 0.97), and enthusiasm questionnaire (*r* = 0.95) had satisfactory reliability indices.

One of the ways the researcher used for deciding on using parametric or non-parametric analysis in a quantitative study is to measure the normality of the data. Table 2 shows the Kolmogorov–Smirnov index, which shows that the distribution of data is not normal (*σ* = 0.000) for any of the variables. The assumption for having a normal set of data is to have a nonsignificant index of K-S, but the output revealed that the data normality rule is violated for all three measured variables in this study, and a nonparametric analysis should be conducted to calculate the possible relationships among the variables.

**Table 2:** Test of normality.

	Kolmogorov–Smirnov^a^		Sig.	Shapiro–Wilk		Sig.
	Statistic	df	Statistic	df
Ambiguity tolerance	0.271	494	0.000	0.774	494	0.000
Teacher burnout	0.301	494	0.000	0.764	494	0.000
Enthusiasm	0.317	494	0.000	0.764	494	0.000

a *Lilliefors significance correction.*

### The First Research Question

The first research question was posed to measure the possible relationship between Chinese teachers’ ambiguity tolerance, burnout, and enthusiasm. Since it was revealed in the previous table that the data are not normal, a non-parametric correlation index (Spearman Rho) was used.

Based on the correlational rules, the greater the amount of the relationship, the stronger the possibility of a significant relationship. As presented in Table 3, the relationship between teachers’ burnout and their ambiguity tolerance is indirect, but significant (*r* = −0.68, *p* = 0.000). Similarly, the relationship between teachers’ burnout and their enthusiasm is indirect, but significant (*r* = −0.62, *p* = 0.000). It can be concluded that the higher the level of teachers’ burnout, the lower their level of ambiguity tolerance and enthusiasm.

**Table 3:** Correlations among teachers’ ambiguity tolerance, enthusiasm, and burnout.

			Ambiguity	Enthusiasm	Burnout
Spearman’s Rho	Ambiguity	Correlation coefficient	1.000	0.592^**^	−0.685^**^
		Sig. (2-tailed)		0.000	0.000
	Enthusiasm	*N*	494	494	494
		Correlation coefficient	0.592^**^	1.000	−0.628^**^
		Sig. (2-tailed)	0.000	.	0.000
	Burnout	*N*	494	494	494
		Correlation coefficient	−0.685^**^	−0.628^**^	1.000
		Sig. (2-tailed)	0.000	0.000	.

**Correlation is significant at the 0.01 level (2-tailed).

### The Second Research Question

The second research question concerns the extent to which Chinese university teachers’ ambiguity tolerance and enthusiasm can predict their level of burnout. This measurement was done by running a multiple regression analysis. The following tables were the output of linear multiple regression analysis including, model summary, ANOVA, and coefficient.

Table 4 provides a model summary for teachers’ ambiguity tolerance, enthusiasm, and burnout. It was shown that the model, which contains the scores of teachers’ ambiguity tolerance and enthusiasm, can explain the amount of variance in the dependent variable (teachers’ burnout). This model can explain 78.20% of the variances in the teachers’ burnout.

**Table 4:** Model summary for teachers’ ambiguity tolerance, enthusiasm, and burnout.

Model	*R*	*R* square	Adjusted *R* square	Std. error of the estimate
1	0.885	0.782	0.782	11.95

Table 5 labeled ANOVA tested the hypothesis that multiple R in the population equals zero (0). The model reached statistical significance (*F* = (2, 491) =883.04, *σ* = 0.000, this really means *p* < 0.05).

**Table 5:** ANOVA for teachers’ ambiguity tolerance, enthusiasm, and burnout.

Model		Sum of squares	df	Mean square	*F*	Sig.
1	Regression	252416.86	2	126208.43	883.04	0.000
	Residual	70175.74	491	142.92		
	Total	322592.60	493			

To measure whether the independent variables (teachers’ ambiguity tolerance and enthusiasm) can predict the dependent variable (teachers’ burnout), the sigma column was studied. As shown in Table 6, both independent variables are significant predictors. Comparing the predictability power, teachers’ ambiguity tolerance (*B* = 0.60) proved to have a higher index.

**Table 6:** Coefficients for teachers’ ambiguity tolerance, enthusiasm, and burnout.

Model		Unstandardized coefficients		Standardized coefficients	*t*	Sig.
		*B*	Std. error	Beta		
1	(Constant)	1.53	1.37		1.11	0.266
	Ambiguity	1.18	0.09	−0.60	12.26	0.000
	Enthusiasm	0.75	0.12	−0.30	6.15	0.000

## Discussion

The present study was an attempt to scrutinize the significant relationship between Chinese university teachers’ burnout, ambiguity tolerance, and enthusiasm level. In addition, it explored the probable predictor role of teachers’ enthusiasm and ambiguity tolerance on their burnout. In the case of ambiguity tolerance and burnout, the correlation is significant but negative. The results of the study showed that incentive and ambiguity of tolerance are the crucial factors in the performance of educators, which need attention in any academic environment. Likewise, the relationship between teachers’ burnout and their enthusiasm is indirect but significant. In other words, the less interested and encouraged the teachers are, the more burnout they may have in their teaching procedure. Indeed, the negative relationship displays that by developing the enthusiasm in educators, their level of burnout will be reduced, which is in agreement with the study by Jennett et al. (2003), who believed that there are many educators all over the world, who may encounter anxiety in their job, which may result in burnout. The notion of managing stress is in direct constructive association with the teachers’ enthusiasm and reduction of burnout (Leiter et al., 2014).

Regarding the second research question, stating that teachers’ enthusiasm and ambiguity tolerance can predict burnout, the findings proved that both variables are predictors of teachers’ burnout. Undeniably, the upshots of the present study are consistent with Kunter et al. (2011) who stated that the educators’ instructing enthusiasm and burnout degrees considering their careers have a relationship. They highlighted that educators with high degrees of burnout will consider themselves to have lower levels of instruction enthusiasm. Furthermore, when they are stimulated and encouraged, they act in the greatest of their capacities in the classroom subsequently their learners become more engaged and cooperate to take part without facing any sense of difficulties to share their ideas. Educators experiencing affective fatigue, disinterest, and reluctance in researching or in making effort in their field, may get unenthusiastic about their career (Kunter et al., 2008). The results are also congruent with the study done by Kasalak and Dağyar (2021) who declared that educators experiencing affective fatigue, disinterest, and reluctance in researching or in making effort in their field, may get unenthusiastic about their career. Moreover, the results support those of Cooke et al. (2013) who made a study on burnout, resilience, and tolerance of ambiguity in Australian general training school administrators. Their findings indicated that ambiguity tolerance, reluctance to show ambiguity, and apprehension were associated with great degrees of burnout. Likewise, the results are in line with McLain et al. (2015) who stated that as a factor of burnout, ambiguity tolerance refers to a significant negative personalization loss factor. Meaning that educators perceiving ambiguous conditions as threat sources can lose engagement with their learners and their career as they constrain interaction with other people to prevent from encountering new complicated conditions (Leiter and Maslach, 2000).

## Conclusion

In particular, outcomes must be utilized to clarify potential executed interferences among educators like the enhancement of their enthusiasm and the success of coaching plans targeted at building it. At the institutional level, interferences must take into consideration elements associated with ambiguity. This research has some significant suggestions for policymakers. In this regard, it is significant for public administrators and teachers to talk about an educator’s function, looking for a practical rearrangement in terms of their burnout in the attributions considering as well as character elements. The findings suggest significant applied implications for teachers. Most importantly, this research arranges for more experimental evidence for the predictable effects of educators’ constructive emotions in the classroom. Even though educator enthusiasm is not universal for all manner of issues in the class (Patrick et al., 2003), it is a strong origin of learners’ behavioral, intellectual, and emotive engagement, innate objective direction, and educational self-effectiveness. Consequently, when learners discern their educators as enthusiastic and energetic, they are more prone to be inherently inspired to study, and engage behaviorally, intellectually, and emotively. It seems that the more enthusiasm educators display in the class, the more effective, involved, and thrilled learners become about education.

Paying attention to educators’ tolerance of ambiguity can affect their psychological and physical well-being and make their behavior more proper in face of ambiguous situations. The tension and apprehension of ambiguity are disappointing and they should be taken into account in training educators. In this respect, educators are recommended to get aware of their reactions to ambiguities, because they should modify their negative responses to the perceived ambiguity when they get aware of how to cope with ambiguous conditions.

People who lack ambiguity tolerance more potentially have negative reactions to ambiguous conditions because it is challenging for them to take risks and correctly judge the lack of data—a state that they consider a “danger and source of inconvenience (Furnham and Marks, 2013). In addition, individuals with low degrees of ambiguity tolerance tend to respond to risk-taking as a kind of tension by delaying, running, suspending, or denying (Furnham and Marks, 2013). As suppliers of a large part of the educational environment, the school faculty will make use of its results. They must place ambiguity tolerance enhancing reasons and strategies in suitable segments of career growth for educators to consider. Through perceiving their ambiguity tolerance, educators can take into account their large number of feasible responses to unforeseen conditions and the way such responses and related options may affect their students in various ways.

Considering the significance of educator enthusiasm for both educators and learners, educator training must particularly attend to nurturing passion and joy in instructing and the topic. In addition, in the daily work of educators, there is a need for a setting that permits keeping enthusiasm. For an instance, keeping away from stressor working situations, namely, the extra workload of responsibilities by the administration and management. As the current research proposed, educator enthusiasm seems to be a significant educator attribute that unveils its influences on their burnout since enthusiasm is built through action and feelings that also improve unity, enhance assets, aid with managing anxiety and alterations, and strengthen and elevate the chances of like activity in the future (Sekerka and Fredrickson, 2013). It is proposed to provide some educator coaching classes which can improve educators’ inspiration for successful and upgraded instructing plans. Furthermore, providing some relevant seminars to lessen the educators’ anxiety and worry is proposed. In these seminars, successful strategies to eliminate weakening anxiety and instructing stress can be presented and instructed to educators. Educators can be also motivated to read some impactful content to enhance their self-esteem and self-confidence, which indispensably lessen their anxiety during the instructional cycle. Furthermore, the research was conducted in 18 provinces of China, which might affect the generalizability of the outcomes. Thus, for further research, it is proposed to include more educators from different regions and university levels. Although the research had a quite large number of subjects as a quantitative study, it is better to conduct similar research with a mixed-method layout, within its qualitative stage, to include an interview as an example, to guarantee how ambiguity tolerance and their beliefs correlated with their burnout.

## Data Availability Statement

The original contributions presented in the study are included in the article/supplementary material, further inquiries can be directed to the corresponding author.

## Ethics Statement

The studies involving human participants were reviewed and approved by Hunan Institute of Technology Academic Ethics Committee. The patients/participants provided their written informed consent to participate in this study.

## Author Contributions

All authors listed have made a substantial, direct, and intellectual contribution to the work and approved it for publication.

## Funding

This study was supported by Research project on teaching reform of colleges and universities in Hunan Province “Exploration and practice of teaching reform of innovation and entrepreneurship course in Colleges and universities from the perspective of accurate supply—Taking entrepreneurship foundation as an example” (No.: HNJG-2021-1088), and Hunan Educational Science Planning Project “Research results in the stage of construction and application of core quality model for teachers of innovation and entrepreneurship education in local applied universities” (No.: XJK21BXJ016).

## Conflict of Interest

The authors declare that the research was conducted in the absence of any commercial or financial relationships that could be construed as a potential conflict of interest.

## Publisher’s Note

All claims expressed in this article are solely those of the authors and do not necessarily represent those of their affiliated organizations, or those of the publisher, the editors and the reviewers. Any product that may be evaluated in this article, or claim that may be made by its manufacturer, is not guaranteed or endorsed by the publisher.
